# Blisters and Calluses from Rowing: Prevalence, Perceptions and Pain Tolerance

**DOI:** 10.3390/medicina58010077

**Published:** 2022-01-05

**Authors:** Joseph N. Grima, Michelle Vella Wood, Nadia Portelli, James N. Grima-Cornish, Daphne Attard, Alfred Gatt, Cynthia Formosa, Dario Cerasola

**Affiliations:** 1 Metamaterials Unit, Faculty of Science, University of Malta, MSD 2080 Msida, Malta; michelle.vella-wood@um.edu.mt (M.V.W.); james.n.grima-cornish@um.edu.mt (J.N.G.-C.); daphne.attard@um.edu.mt (D.A.); 2 Department of Chemistry, Faculty of Science, University of Malta, MSD 2080 Msida, Malta; 3 Junior College Physics, Junior College, University of Malta, MSD 2080 Msida, Malta; nadia.portelli@um.edu.mt; 4 Department of Podiatry, Faculty of Health Science, University of Malta, MSD 2080 Msida, Malta; alfred.gatt@um.edu.mt (A.G.); cynthia.formosa@um.edu.mt (C.F.); 5 Sport and Exercise Sciences Research Unit, Department of Psychology, Educational Science and Human Movement, University of Palermo, Via Francesco Spallitta, 52, 90141 Palermo, Italy; dario.cerasola@unipa.it

**Keywords:** rowing, blisters, calluses, dermatology, hands, pain, tolerance, acceptance

## Abstract

*Background and Objectives:* Rowing is a sport that involves constant gripping, pulling/pushing, and rotational movements of the hands, in a cyclic periodic manner with every stroke, with hundreds of strokes being taken within a short period of time. Dermatological issues on rowers’ hands (fingers and palms) in the form of blisters and calluses are common knowledge within the community, but their prevalence and the rower’s perceptions and pain tolerance to them has never been systematically evaluated. This work addresses these lacunae. *Materials and Methods:* Analysis of data collected from a survey on a sample of competitive (117) and noncompetitive rowers (28) who row on-water (total 145). *Results:* It was found that approximately 69% of rowers participating in this study have calluses on their hands for most of their time (considered by them as not painful). The incidence of blisters was found to be lower (but perceived as more painful). Their incidence was found to be fairly independent of the frequency and intensity of training, but they seem to affect most rowers equally at the beginning of season or during a change of position (nonconditioned hands). Blisters and calluses were reported to be mainly located on the proximal phalanges and metacarpo-phalangeal joint area of both hands, i.e., on the lower parts of the fingers and the upper inner palms. *Conclusions:* Rowers demonstrated a sense of acceptance of these dermatological issues, even a sense of pride in what they represent. The incidence of blisters becoming infected was estimated to be so low that most rowers would not have encountered such serious, albeit rare, consequences.

## 1. Introduction

Rowing is a whole-body exercise that is practiced in the modern world as a leisurely activity and a sport. With the establishment in 1892 of the World Rowing Federation (*Fédération Internationale des Sociétés d’Aviron* (FISA)), the world governing body of the sport of rowing, rowing as a sport gained more uniformity and regulations to enhance its universality. In fact, World Rowing, through its rule book [1], now sets the rules and regulations for the practice of rowing at international level for its on-water (coastal and flat-water) regattas and for indoor rowing (practiced on rowing ergometers that emulate the rowing action). As a result, with the exception of the historical regattas rowed on traditional local boats, the sport is normally practiced on highly standardized sliding-seat racing boats, or shells, which are regulated in terms of dimensions and weight. On these boats, the rower has her/his feet anchored on a fixed foot rest, and sits on a sliding seat facing the rear of the boat. This seat slides backwards and forwards, thus allowing the rower to utilize leg strength as optimally as possible.

**Figure 1:**
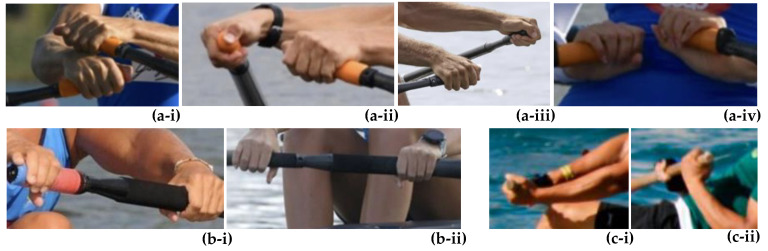
Images showing some of the hand movements in rowing, where (**a**) shows images of the standard sculling stroke, (**b**) shows the hand grip in standard sweep rowing, whilst (**c**) shows the reverse grip style of holding the oar, which is not unusual in traditional fixed-seat rowing.

On water, rowers can either row holding one oar with both hands (sweep rowing) or row holding a pair of oars, one in each hand (sculling). From the perspective of the hand, the rowing action involves constant “gripping” of the oar (with one or two hands), “pulling” actions, and a “rotation” (feathering) of the oar with every stroke during the drive phase, as well as a less demanding “pushing” and rotation during the recovery phase of the stroke, as described in Figure 1.

The “stroke” on a standard rowing ergometer is simpler in form as the rotational feathering action is not required, i.e., it only involves “gripping” and “pulling” in the drive phase.

**Figure 2:**
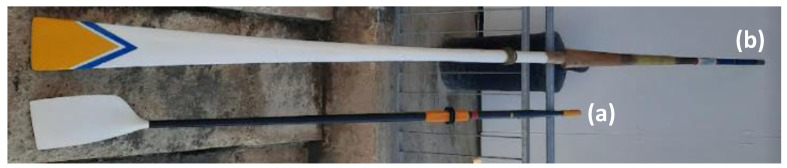
Oars, where (**a**) shows a typical modern oar used in sculling whilst (**b**) shows a typical, more traditional, wooden oar, still in use in traditional fixed-seat rowing races (in this case, a sweep oar used in the Maltese traditional regatta). The difference in size, weight, and difficulty in handing and feathering between these two types of oars should be appreciated.

Typical oars used for sculling are around 284 cm–290 cm in length, whilst oars used for sweep rowing are typically longer, *circa* 370 cm–376 cm in length. Oars are now typically all made from composite materials, which provide excellent weight-to-strength ratio and permit mass production, with the more common shape of the “blade” being the hatchet/cleaver blade style, first introduced in 1992, which is shown in Figure 2a. The oars used in fixed-seat boats, mainly seen in traditional events with a historical context, such as the Maltese Traditional Regattas and in the Cornish County Championships (UK), are typically heavier (i.e., more difficult to handle and feather), designed in a more traditional manner, and made of wood as shown in Figure 2. Note that in such traditional rowing, the use of a “reverse grip” for holding the oar is not uncommon (see Figure 1c, to be compared with the sweep “standard grip” in Figure 1b).

In the on-water World Rowing races, the standard rowing distance is 2000 m, typically lasting five and a half to eight minutes. Shorter distances of 1000 m are also not uncommon, particularly for master athletes. Coastal rowing races are normally either “endurance races”, which are much longer, with typical events being four to ten kilometers long, or “beach sprints”, which are much shorter races and start and finish from a beach. It is well known that the standard 2000 m race is “one of the most physically demanding efforts in the world of sport”, which “is not only physically demanding, but mentally exhausting as well” [2]. Rowing is therefore not an easy sport to practice, and rowers are known to endure “unthinkable amounts of pain” [3] and still keep rowing.

In 2020, the International Association for the Study of Pain (IASP) redefined pain as “An unpleasant sensory and emotional experience associated with, or resembling that associated with, actual or potential tissue damage” [4]. Pain indicates, by giving signals, when harm is being done to bodily systems, so that a response stops further damage. Even though pain is a protective mechanism, all through evolution survival depended on tolerating painful stimuli, and in most sports, pain is inevitable. To obtain desirable results, most athletes have learnt to either tolerate pain or developed strategies to counteract it. Practicing sports activities, especially at a high level, induces pain [5], and having the skills to tolerate or overcome this pain leads to success or failure in sports [6]. Pain during exercise also induces fatigue, which, according to Gandevia [7], is a “reduction in maximal voluntary muscle force. It may arise not only because of peripheral changes at the level of the muscle, but also because the central nervous system fails to drive the motor neurons adequately”. When dealing with pain, research suggests that athletes tend to use “acceptance” as a form of cognitive strategy [8,9].

Pioneering work published in 2003 by Ord and Gijsbers [10] looked in detail at pain thresholds and tolerances of competitive rowers and their use of spontaneous self-generated pain-coping strategies. A more recent study by Cohen et al. (2009) [11] adopted a similar protocol as that described by Ord and Gijsbers [10] in their study about pain tolerance and thresholds of rowers, to evaluate pain thresholds for individual and group rowing. The authors postulated that when rowers trained as a group, their pain threshold was more elevated than when rowing individually due to an elevated “opioidergic effect”, similar to a runners’ high, induced by a sense of bonding when training with trusted team members. Such synchronized activity when working as a group, as demonstrated when rowing together, releases endorphins, which anecdotally might be called “a feel-good factor”, and this has the effect of increasing the pain thresholds when rowing. In agreement, Davis et al. (2015) [12], argue that a sense of goodness, which might also be referred to as a “flow” state, is achieved when athletes train together due to the release of endorphins secreted when the group is working together, synchronized and bonded as one entity. Such a state leads to higher thresholds when measured after training.

Although prominence is normally given to lower-back and knee pain induced from rowing, injuries from rowing are not limited to musculoskeletal issues. In particular, during rowing races and/or training, athletes require high levels of both strength and aerobic power [13,14]. Typical maximal anaerobic power scores have been estimated at 900–1100 Watt (W) for heavyweight men and 650–800 W for lightweight men [15]. In rowing races, athletes tend to adopt a positive race profile [16,17,18,19], which could be attributed to the athletes’ initial powerful phase. In fact, according to Steinacker (1993), forces of 1000–1500 Newton (N) are typically needed in the start of the race, 600–800 N in the start phase, 500–700 N during the race, and 600–700 N for the finish [20]. Thus, the practice of rowing, which typically involves hundreds of strokes in a very short time where rowers grip and move the oars with such magnitudes of power, inevitably results in the hands of the rowers being subjected to strong tensile and shear forces, as well as friction, of a very high magnitude. This repeated friction between hands and oars exposes the anterior part of the palms and fingers to stressful shear forces, which may cause separation of skin layers and the development of friction blisters. [21,22,23,24,25,26]. “Friction blisters” (in this paper, referred to simply as “blisters”) are the result of excessive frictional trauma to areas of the skin [27]. Practitioners who work in the sports of rowing know too well that rowers typically suffer from hand blisters, and, as well stated by Karlson (2000), “most rowers merely tolerate blisters as a necessary evil that will resolve as the skin adapts” [21]. Mokha et al. [26], in their work “Dermatoses in Rowers”, describe blisters as “painful vesicles and bullae on anterior surfaces of the palm and fingers”. The authors attribute these blisters mainly to the friction between hand and oar during feathering. They also state that (in sweep rowing) the hand closest to the rigger develops blisters on the palm, while the other hand, which grips the oar in a hook-like manner, develops blisters on the fingers. The authors also suggest that the tendency to develop multiple blisters can impact on hand placement on the oar due to the pain. The treatment known as “paring” by draining while leaving the roof of the blister untouched as well as taping or bandaging during rowing to avoid irritating them further is well known and described by various authors [22,26,28]. Additionally, switching between different oar handle textures or types should be avoided.

The stratum corneum, the outer horny layer of the skin, can become thickened or callused, also termed “hyperkeratosis” (this paper, referred to simply as “callus”). Whilst being a protective mechanism to shield underlying structures from harmful repetitive stress, as the hyperkeratosis thickens, it can become a source of pain in itself. In other words, over time, repeated skin distress leads to the formation of protective hard skin calluses. [22] Mokha et al. [26] describe the rowing-induced calluses as yellowish “hypertrophic and thickened skin”, which serves to provide relief from excessive friction. This is hence an adaptation that rowers normally desire [22]. Thus, it is not usual for rowers to seek treatment for calluses, though rowers typically trim or file them if the calluses become excessive [22,28].

Blisters and calluses in sports are not unique to rowers. Blisters are reported in various sporting events, for example, marathon running, with an incidence of up to 25% in athletes’ feet [29]. A large national study of sport- and physical exercise-related injuries in the United Kingdom reported an occurrence of 2.2% for “tenderness, swelling, blisters” out of the 1429 respondents that reported being injured in any way. [30]. No detail on the particular sport practiced in these cases was given. More specifically on hand skin injuries, a study by Smith and Krabak (2002) describes how 19.5% of participants of the 1998 Tug of War world championships reported “Abrasion/burn/blister” injuries. [31] The authors commented on the similarity between Tug of War and sweep rowing when it came to injuries and also reported an observation that blisters appear to be “quite common”. Blister occurrence was also reported on in the sport of fencing, where 48%–65% of treatments needed during competitions are attributed to blistering [32,33,34], as well as in the related yet distinct sports of canoeing and kayaking. A recent study by Feher reported that a quarter of participants in canoeing marathons get blisters [35]. Dragon boating (an activity that is more similar to kayaking than to rowing) seems to be more inducive to hand blisters, with as many as 78.9% of dragon boaters in a study reporting blisters. [36]

Despite the fact that rowing causes blisters and calluses on the palm of the hand and fingers, it is common knowledge that, in general, training generally continues, possibly exacerbating the issue and increasing the possibility of infection [22]. This problem may be further aggravated when the rowers decline the use of gloves, which are perceived as hindering the real feel of the oar [21]. Notwithstanding such interesting observations, and the availability of various online publications aimed at rowers that discuss rowing blisters and suggest ways to care for them [37,38,39,40], no study seems to have looked into this issue in a more quantitative manner. In fact, to our knowledge, although the prevalence of blisters and calluses is generally regarded as high in active participants, there is no reported quantitative research to systematically evaluate the prevalence of blisters and calluses, where they occur, the manner in which rowers react to them, and the impact these blisters and calluses may have on rowers (e.g., on their performance in the sport and/or on other aspects of their life).

In view of this, the present study is intended to form initial evaluations about trends, opinions, and frequencies obtained quantitatively about blisters and calluses of on-water rowers. The research questions in this study are envisioned to descriptively investigate how these dermatological conditions affect water rowers’ hands, mainly their fingers and palms, inquire about the frequency of their prevalence, causes, and care. Another intent of this study is to inquire about the rowers’ perception of pain while rowing on water and how these dermatological conditions affect the rowers’ life and identity.

## 2. Materials and Methods

### 2.1. Participants

The researchers used mainly a multistage clustering [41] procedure to procure participants. Different social media groups or other rowing groups and clubs were identified, and an online questionnaire was sent to the group/club participants.

The inclusion criteria to participate in this study were that the participants had to be active participants in the sport, practicing on-water rowing (i.e., not just ergometer rowing), and over 18 years of age. There were 145 such participants in this survey, hailing from different countries, although nationality was not a criterion for selection. The 145 responses were distributed as follows: 29% from the US, 30% from the UK, and the rest distributed among another 20 countries. Different levels of rowers were identified from the sample collected, although rowing level was not a selection criterion. Twenty-eight (28) of the 145 participants were amateur rowers, 65 were regional/club-level rowers, while 56 out of the 145 participants were national/international-level rowers.

### 2.2. Epistemology, Ontology, and Methods

Regarding ontology and epistemology, this research is built on the scientific paradigm because it was designed to obtain knowledge that “seeks predictions and generalizations” thus, methods to generate quantitative data [42]. Adopting this scientific approach, methodology was based on the ontological view that “a discoverable reality exists independently of the researcher” [43]. The phenomenon being studied was external to the researchers, who looked at it in an objective manner. This led the researchers to adopt a quantitative method of gathering data through a series of closed-ended questions, as much as possible.

After obtaining ethical approval (University of Malta Research Ethics Committee, through the Faculty of Science Research Ethics Committee), participants were recruited through social media rowing-focused groups, rowing clubs, and word of mouth. They gained access to an online set of questions after consenting to participate in the study, and they were asked to answer these questions as truthfully as possible.

### 2.3. The Instrument

The preferred instrument used was a survey developed specifically for this study. This method was adopted so that the collection of data could easily and efficiently answer the research questions. The survey data was cross-sectional in nature [41], with the data being collected within days from the distribution of the online survey. Such a distribution was the mostly likely method of data collection reasoned to reach a high number of rowers from the international population. It was the most efficient, time-effective, and convenient way to obtain data from different groups of rowers in a short time interval. The scope of the study was explained to the respondents, who participated on a voluntary basis. They were also told that responses were confidential

The first part of the questionnaire consisted of demographic information, for example, age groups, whether they row or scull, years practicing the sport, and the intensity and durations of their training. Subsequent parts of the instrument focused on blister and callus formation, the ability to function with blisters and calluses, related pain, and effect of blisters and calluses on daily routines. The survey mostly consisted of multiple-item questions with Likert scales (e.g., 1 (not painful) to 5 (very painful) or 1 (strongly agree to 5 (strongly disagree)), or else “multiple-choice” questions, where the participants were given a variety of answers to choose from obtained from literature and experts in the field, generally giving the possibility to add their own response if they felt the need. For some questions, the respondent could only choose one reply (single-choice questions), whilst in others, they could choose a number of replies (multichoice questions), as noted in the captions of the tables in Section 3.

### 2.4. Data Analysis

Since the intent of this study was to look for trends within the data and to form initial evaluations, a descriptive statistical analysis [44] was carried out by an experiened researcher who looked at such measures within the data available. Such trends emerging from this analysis will be useful to inform the researchers about common trends or links between the variables, which may be later used in future studies to look for relationships and associations. The responses were tabulated and analyzed to gauge distribution according to the rowers’ competitive level (further explained in Section 3.1.1), and, unless otherwise stated, the responses were grouped into three sets:

**Category I:** Rowers who do not compete (i.e., only row for fun or claim that they train seriously but do not compete);

**Category II:** Rowers who compete at regional and/or club level;

**Category III:** Rowers who compete at international and/or national level.

## 3. Results

### 3.1. Responses

#### 3.1.1. General Distribution of the Responses

All 145 rowers (age: mean = 43.8 years, standard deviation = 12.6 years, gender: 48.3% male, 51.7% female) who participated in the study were active rowers and devoted time to the practice of the sport. Some of them practiced more than one form of rowing. As shown in Table 1, when asked how many hours they devoted weekly to each type of rowing, the majority of the respondents reported spending hours of their time rowing indoors on an ergometer and on water. The vast majority of respondents, 89%, reported using a rowing ergometer, while 82% of rowers reported spending time on sliding-seat sculling, with 35% and 39% reported spending their training time in sliding-seat sweep rowing, using right- and left-side oars, respectively. A number of rowers, 15%, reported that they row on fixed-seat boats. The distribution of training hours is shown in Table 1 below and is consistent with the fact that sliding-seat rowing is much more widespread than fixed-seat rowing, and that training on a rowing ergometer is routine for most on-water rowers.

As noted in Table 2, more than half of the 145 responses (51.72%) reported that they had been rowing for over 10 years, while 40.69% had been in the sport between 3 and 10 years. Only 7.59% had been involved for less than 3 years, all of whom had rowed for at least one year. Moreover, as noted in Table 3, nine (9) of the rowers, or 6.21%, claimed to be competitors at an international level. Another 47, or 32.41%, claimed to compete at up to national level, while 61 (42.07%) competed at regional or club level. However, the remaining 19.31% did not compete and rowed “just for fun” (9 rowers, 6.21%) or considered themselves as “a rower who trains seriously but does not compete” (19 rowers, 13.10%). This information justifies the applied categorization criteria based on why the respondents row, particularly in view of the fact that as shown in Table 4, the categories of rowers rowing competitively and noncompetitively seem to be similarly distributed in terms of years of experience, with the most experienced rowers being those rowing at national or international level (64.29% of whom had rowed for more than 10 years).

When one re-analyzes the data on how much time the rowers report as spending on-water, one finds that the rowers who row and compete at national and/or international level (Category III) spend considerably more time rowing on water when compared to the other categories, particularly those rowing merely for fun (Category I, see Table 5). Rowers were also asked to rate their perceived intensity when they row, from a scale of 1 to 5, with 5 being the hardest. As shown in Table 6, most rowers who row to compete (Category II and III), tend to perceive that they row at a harder intensity compared to those who row for fun (Category I). In fact, around two out of three rowers who compete at club, regional, national, or international level ranked their rowing intensity as 4 out 5, and around 10% of rowers competing at national or international level stated that their rowing intensity is 5 out 5. In contrast, more than half of rowers who do not compete, and row solely for their personal enjoyment, ranked their intensity at 3 out of 5 or less.

#### 3.1.2. Prevalence of Blisters and Calluses Caused as a Direct Result of Rowing, Their Perceived Cause and Their Location

As shown in Table 7, the study confirmed that regular rowing results in hand blisters and calluses, with calluses being much more of a common occurrence than blisters. In fact, 15% said they got blisters regularly, but the vast majority (over 70%) reported that they get blisters “occasionally or rarely”, whilst almost the same large amount (69%) reported getting calluses regularly (i.e., more than 50% of the time). Overall, only three rowers (2%) reported never getting blisters, while 11% stated that they got blisters when they first started rowing but not anymore. Blisters are a recent memory for most. With the exception of the three who claimed never to get blisters, 24 had blisters on their hands at the time they responded to the questions, while another 89 had them in the 12 months prior. Just 29 had blisters over a year before. With respect to calluses, one never had any, while 12 last had calluses over a year prior. Forty-four (44) had calluses within the last year, while 88 responded that they had calluses “now”.

An interesting feature that emerges here is that, despite the aforementioned differences in the frequency and intensity of training, the distribution of responses in terms of the incidence of blisters was consistent among Categories I, II, and III. This indicates that there is not much difference in the prevalence of blisters and calluses between the three categories of rowers, with rowers rowing at a perceived low intensity and less frequently reporting similar incidence to those who train more frequently and intensely.

To appreciate the extent to which rowing causes blisters and calluses, the data in Table 7 should be compared with the data in Table 8, which summarizes the responses that respondents provided to a question whether they get blisters or calluses from any other non-rowing-related activities. This comparison clearly confirms that the main cause of the rowers’ hand blisters and calluses is the rowing itself!

The replies provided by the rowers when asked to describe where their rowing-caused blisters and calluses are typically located, and what causes them, are reported in Table 9 and Table 10, respectively. The main suspected cause of these blisters and calluses, irrespective of whether the rower rowed for fun or to compete at national/international level, was thought to be unconditioned hands at the start of the season (50%+ of rowers share this opinion), or when rowers change oar or position (57% of rowers who row at national/international level have this opinion). This perceived link between unconditioned hands and the onset of blisters and calluses may explain the finding that the occurrence or otherwise of blisters and calluses seems to be independent of the frequency or intensity of rowing, since, it seems, blisters and calluses will probably and unavoidably form with the onset of the rowing season (or rowing in a new position). Other rather interesting responses, suggested by the respondents, included wet hands, salt from the sea, sensitive skin, and slippery handles (such as plastic handles in rain). Environmental conditions may also play a role, and one rower stated that the issue seems to exacerbate “when the water is rough and I have to grip harder or steer to boat with one hand more forcefully”. (For this question, respondents could choose from a set of pre-suggested causes, or propose their own.)

In terms of location (see Table 9), our data suggest that the most common location of calluses and blisters that result from rowing are the lower parts of the fingers and upper palm of the hand, followed closely by the remaining parts of the fingers.

#### 3.1.3. Pain Rating, Attitudes and Perceptions about Blisters and Calluses

When asked about their opinions and perception of blisters and calluses (see Table 11), almost all rowers agree, or strongly agree, that they are “Inevitable: Most rowers get them”. This opinion was practically equally shared by all three categories of rowers. A good number of rowers also think that blisters are painful and/or ugly. In fact, it is only a few rowers from the Category I who practice rowing for fun rather than competition who seemed to strongly dissent from this perception.

However, as shown in Table 12, the rowers do not seem too concerned about their blisters and calluses, probably because they would have become accustomed to them. For example, when asked to rank their blister and callus pain perception on a five-point Likert Scale, with 1 being not painful to 5 being extremely painful, the overall majority (32.42%) gave a pain perception rating level of 3 regarding the pain originating from blisters, with the club/regional-level rowers (Category II) forming the vast majority (43.40%) of this rating. Surprisingly, Category I rowers were the group who mostly claimed to not feel pain from blisters. The absolute majority of respondents did not report any pain from calluses and rated it as level 1. Note that the data in Table 12 only considered responses of those rowers who actually had experienced blisters/calluses.

It is also interesting to note that, despite what is reported in Table 8 and Table 9, few rowers seem to think that blisters and calluses have an effect on their ability to row well, their work, or their personal life (see Table 13). Moreover, the vast majority of the respondents stated they keep rowing despite the blisters, with the main reason being that they have “learnt to accept them’ (71.43%). Around half also responded that they continued due to their love of rowing. Just four (4) would stop rowing until the blisters heal. This distribution was consistent among the three competitive groups (see Table 14). It is interesting to note that very few rowers keep rowing due to fear of losing their seat on the boat. Other motives determine their willingness to continue rowing despite the pain as discussed in more detail below.

The manner in how rowers deal (or rather, do not deal) with their blisters and calluses is also noteworthy to highlight. As shown in Table 15, first and foremost, very few rowers seek medical advice or visit a health/medical professional when they get blisters or calluses. Instead, the vast majority either just ignore them or drain them/remove them themselves. Here, rowers, particularly rowers in Category III, seem to realize that blisters (which are effectively open wounds) should not be left exposed, and the bulk (as much as 50% of Category III rowers) cover them with tape. Gloves are however unpopular with Category III rowers, although c. 25% of rowers in Category I and II admit to using them when they have blisters. Notwithstanding this, the incidence where infection does in fact occur seems to be fairly low (see Table 16), and rowers report that these issues tend to solve themselves on their own, with blisters eventually becoming calluses, which are less painful. It is thus not clear whether rowers use tape to cover blisters simply to be able to continue rowing, or to protect themselves from infection, but this practice is probably helping to ensure that blisters do not get infected.

Another really interesting aspect that results from this research is that despite what is reported in Table 11, a good number of rowers seem to be actually rather proud of their blisters and calluses (see Table 17). In fact, approximately a third of all the rowers (38%) considered blisters and calluses to be a sign of hard work, 34% were neutral, while 27% disagreed. Almost half (48%) identified with the statement that blisters and calluses were “something to be proud of: they show I AM A ROWER!!”, 28% were neutral, while 24% disagreed with the statement.

## 4. Discussion

This study has drawn some very valid and novel conclusions about the behavior of rowers. First and foremost, it confirmed quantitatively most that was known qualitatively, i.e., that, as stated in a previous publication by [21], the “hands of rowers are highly susceptible to blisters” and that “most rowers merely tolerate blisters as a necessary evil that will resolve as the skin adapts.” [21]. In fact, most of the participants have admitted that they are prone to blisters and calluses, with only 2.07% stating that they never had blisters and 1.38% that they never had calluses. What is the most remarkable, and previously undocumented, is the magnitude of the problem, with almost 81.38% acknowledging suffering from blisters and calluses on different degrees of regularity. Such a high prevalence in hand blisters and calluses puts rowing as one of the sports with the higher incidences of such skin conditions [31,32,33,34,35].

Also remarkable is the rowers’ attitude. The rowers are willing to withstand the pain (confirmed through this study), even despite knowing there is the possible exposure to infection, much highlighted by sports medical practitioners [23], even ones in the field of rowing, with Karlson (2000) [21] stating that “A few may get secondary infections, which often require oral antibiotic treatment. More serious infection is rare” [21] with Rumball et al. (2005) [22] stating that “blisters … may lead to infection (‘sausage fingers’) if not cared for properly in the acute stages.” [22]. Most participants admitted that they would either do nothing (28.57%) to cure blisters or self-medicate (44.44% draining; 43.65% taping), as suggested in various online publications [37,38,39,40]. Here, it must be said that since our research found such a low incidence of blisters being infected, it could well be that most rowers who participated in the study may be oblivious to the fact that infection may be a real threat. In fact, it may be extrapolated from the data collated in this study that, given the low incidence of such infections, most rowers will never experience infected blisters either on themselves or on a fellow member of their crew. It must, however, be said that the scientific rowing literature reported that there is a risk, albeit low, that there could be serious consequences if blisters get infected, and this should not be overlooked. A respondent made this very clear though a very poignant comment “My son was hospitalized for a rowing related hand infection. I.V. Meds for days. He rowed at a national level.”

This research shows that this population not only accepts blisters and calluses, but also accepts the fact that they are part of the rowing lexicon. Nearly all of the participants agree that blisters and calluses are inevitable (93.10%), and almost half (47.58%) of the respondents agree that blisters and calluses are something to be proud of, as they demonstrate that they form part of the rowing culture. However, less than half of the rowers in this study agree that blisters are a sign of hard work. Rowers have admitted that the formation of blisters may be attributed to “gripping too much” (35.71%), when they change the oars or row in a different position (14.29%), or “unconditioned hands” (50.00%), for example, at the start of the season. Past scholarly studies in fact espouse the formation of blisters with how the oar is handled [21]. Future research can also correlate the type of oars used and the type of handling with blister formation.

Another unique finding from this study is that the discomfort or pain from blisters and calluses does not affect rowing performance and task completion, with 74.10% admitting that blisters and calluses have no effect or a minor effect on rowing itself, with the majority of rowers (93.10%) admitting that blisters and calluses are inevitable and part and parcel of a rower’s life. Most participants have also agreed that they do not think that blisters or calluses have an effect on the quality of their personal life (76.98%), their work (81.29%), leisurely activities (80.60%), relationship with friends (92.80%), or the image of themselves (92.80%), even if 39.40% admit that blisters and calluses are “ugly”.

This research has exposed the finding that the majority of the rowers (76.26%), who are also active competitors, claimed that having both blisters and calluses would not stop them from rowing, indicating that they would still row even when they perceive pain or discomfort, as they themselves admitted to feeling, albeit at different levels. This finding is similar to the interesting finding that emerged from research carried out by Hoffmann and Krouse [45], who asked participants whether they would stop practicing the sport (running) if it proved to be detrimental to their health. Of the athletes, 74.1% admitted that they would not stop their activity, similar to the finding in this study. The fact that the rowers do not stop rowing, despite having blisters and calluses, indicates that they have learnt to cope well with this stressor. Coping in sports refers to mechanisms that athletes adopt to manage stressors or stressful situations to ameliorate their relationship with the said stressors while practicing their sport. Lazarus and Folkman [46] define coping “as a constantly changing cognitive, behavioral, and affective process individuals use to manage stressors that are appraised as taxing or exceeding their resources”. These mechanisms, which might be cognitive in nature, can also augment the pain tolerance of the athletes. Roebuck et al. [47] argue that an elevated pain tolerance in the athletic population is achieved by the use of cognitive coping mechanisms that are spontaneous and involuntary. In their study, Ord and Gijsbers [10] found that the elite rowers could tolerate pain induced by sphygmomanometer pressure for a much longer time period than the controls could. These rowers attributed their higher tolerance to using multiple high-level cognitive methods to control pain sensations. A salient theme that emerged from this study is that almost all of the participating rowers (76.26%) admitted that they would keep rowing even with these dermatological ailments, because they have learnt to accept them. Rowers are indeed aware of the fact that blisters and calluses are going to be present throughout the season and used acceptance as a mechanism to deal with the pain and discomfort. Acknowledging and accepting a stressor is a cognitive coping strategy [6] used by sports practitioners mostly when they cope directly with the stressor in order to deal with it. The participants did not turn away from the stressor (pain) or use methods to avoid it (most do not even take measures against the pain and likely infection caused by blisters) but rather affronted the stressor and accepted its presence. Carver et al. [8] argue that acceptance occurs during secondary appraisal and is a functional coping response in which the stressor is something that is “accommodated to” since it cannot be changed.

Cognitive strategies to manage pain and enhance tolerance during exercise were also described in a study carried out by Simpson et al. [48], where athletes described that they employed mental skills to manage adversities such as fatigue and pain to maintain a good performance. Tezars et al. [49] in fact also discuss that there is a positive relationship between pain tolerance and acceptance. Once the discomfort is accepted, discomfort can be tolerated better. Carver et al. [8] believe that acceptance is a strategy used when there is no other alternative and by engaging in such a strategy, the rowers are in fact dealing with the stressor to ameliorate the situation. Costa and Pinto-Gouveia [50] argue that acceptance of the stressor, in this case pain and discomfort originating mostly from blisters, makes sense since it is useless fighting the inevitable when energetic resources can be spent in a more fruitful manner.

Past studies have shown that athletes who adopted this cognitive method could then focus inward on themselves to complete the task without any form of distraction. Most of the rowers (76.3%) in this study were similar to the elite runners described by Morgan and Pollok (1984) [51] who used associative strategies and read corporeal signals without ignoring what their body was telling them. The rowers admitted that they accepted the blisters, calluses, and related pain, thus converging their cognitions inwards and forming a task-relevant (internal) association as described by Stevinson [52]. Other past literature [10] has shown that the pain tolerances of rowers are quite elevated when compared to controls, although there was no distinction between pain thresholds [53,54]. (The greatest amount of pain an individual demonstrates willingness to assent to is called pain tolerance [55], while pain threshold is defined as the minimum intensity of a stimulus that is perceived to be painful [53].) This concurs with our finding that fun and competitive rowers rate pain due to blisters at approximately the same levels, indicating that they have the same thresholds. In the case of this study however, almost all the athletes admitted that they would continue rowing despite the blister pain, so no significant pain tolerance distinction between the groups could be exposed. Future research might try to analyze this further, by adopting different protocols to assess pain tolerance between the groups.

Having a solid control of situations that cause stress indicates a good level of mental toughness. There are various definitions of mental toughness, one of them being the ability to make use of coping strategies effectively [56]. Extant research has been shown to bind the construct of mental toughness with effective coping [57,58,59], which then leads to successful task completion despite having to deal with stressors like pain. In the case of the participants in this study, mental toughness was demonstrated by the willingness of the rowers to continue rowing despite the formation of blisters and calluses and also by adopting a strong cognitive strategy to enable them to complete their task.

The fact that all groups admitted that they would carry rowing on despite the pain from blisters reveals that most (76.3%) of the rowers, as a population, demonstrated that they have a superior pain tolerance. Tesarz et al. [49] revealed that an elevated pain tolerance is also related to a long-standing training regime that may alter pain perception. In reality, most (51.7%) of the participant rowers have been rowing for more than 10 years and 40.7% have been rowing between 3 and 10 years, and this longevity in the sport might be a contributing factor that affected their tolerances. It must however also be said that the study also suggests that painful blisters typically occur at the start of the rowing season, which typically is characterized by long steady-state rows, which are likely to be more tolerable by rowers with blistered hands when compared to high-intensity power-burst workouts, such as interval training or “starts”. Additionally, it is more than likely that most members of the crew on the same shell would be afflicted by blisters of some degree, i.e., no individual rower would feel under the pressure of letting down fellow crew members.

Another significant finding in the case of rowers in this study was the admission of rowers that their willingness to continue rowing despite the blisters and calluses was also related to their affinity towards the sport. Results show that 45.3% of the participant rowers admitted that they would not stop as they “simply love the sport”. Elite runners described by Morgan and Pollok [51] showed a similar trait when they admitted that they enjoyed the feel-good factor the sport rewarded them with. Future research may be aimed at finding the reasons why participants have this passion for rowing.

Before concluding, it is important to mention some issues that merit further study. For example, in view of the high prevalence of blisters and calluses encountered by these athletes, further research is required in order to address the etiology of these lesions. Such research should also look into ergonomics and equipment design. Furthermore, it is not clear how the majority of the respondents of the present study say that blisters do not affect their rowing, when extant research has shown that blisters might be the deciding factor of an athletic competition if it results in reduced performance or incapacity for a key person at a critical time [23], and can also cause intense pain [24]. Indeed, 10.34% strongly agree and 45.52% of the participants agree that they perceive some sort of pain or discomfort from blisters acquired during rowing, of which 5.52% perceive this pain as extremely painful. Future research can reveal the reasons why very few rowers deem rowing with blisters and calluses as being extremely painful and why a majority of amateur rowers find it not painful at all.

Another aspect that deserves to be looked into more closely is the very interesting finding that most of the participants who deemed blisters as not painful (level 1) were noncompetitive athletes (29.17%, as opposed to Club level—7.55% and National/International level—10.20%), while most club/regional and national/international athletes deemed blisters as only being reasonably painful (3 on a scale of 1 to 5). This might be due to different training regimes and expectations from athletes of different levels. In fact, our results show that national and international rowers train at a much higher intensity than the fun rowers and club/regional-level athletes do since they participate in high-level competitions and may need to give maximum effort, which might not be the case when rowing for fun and health. For example, when the training intensity reaches level 4 on a scale of 1 to 5, only 35.7% of the Category I rowers admitted to training at that level, in contrast to 67.2% of club/regional-level (Category II) rowers and 64.3% of national/international (Category III) rowers. A limitation of this finding is that the athletes were not assessed on the same parameters to rate pain, such as using the same protocol to assess the pain induced by rowing in the same conditions.

Finally, it is important to highlight some of the strengths and limitations of this work. An important limitation is that the study was always conducted remotely and the respondents could not interact with the researchers carrying out the study. This could lead to some inaccuracies stemming from misunderstanding questions. Moreover, the method used made it impossible to decipher whether the respondents were answering questions truthfully. On a more positive note, this work has finally addressed the issue of hand blisters and calluses in a quantitative manner, and was able to confirm various opinions put forward by others. It also brings to the limelight what rowers really think of their blisters and calluses and how they deal (or rather do not deal) with them. Such study, to our knowledge, had never been carried out before. The present study could also form the basis for further, more focused studies, such as whether the forced extended breaks from rowing caused by COVID-19 lockdowns could have aggravated or alleviated rowing injuries, including skin conditions. Further studies could also look into why so few rowers seem to suffer from infections when they have blisters on their hands. It would also be useful to look into special populations, such as junior athletes, para athletes, as well athletes with specific medical conditions such as diabetes. In addition, the participants in the present study tended to row in more than one position, making it futile to attempt to relate location of blisters and calluses to the manner how the oars are held. As a result, in a future study, it would be useful to focus on rowers who only practice sweep rowing, or who only scull. It would also be interesting to examine athletes who exclusively practice fixed-seat rowing so as to assess whether they are more prone to blisters and calluses. Moreover, the future study could ask more specific questions, such as how the rowers work-around blisters to be able to continue training, or how prepared they would be to reduce the intensity of rowing training if they, or fellow crew members, are afflicted by painful blisters.

## 5. Conclusions

In conclusion, this study has quantified the prevalence of blisters in rowers, which seems to be independent of the extent and intensity of rowing, and demonstrates that although there may be pain associated with them, for most rowers, the passion for rowing is stronger than the pain itself. More specifically, it was estimated that around 69% of rowers are regularly sporting calluses on their hands, which they generally classify as not painful, whilst the prevalence of blisters is less pronounced. It was shown that the more common locations for these blisters and calluses are the lower parts of fingers and upper part of the palm.

This work has found that the participants claim that blisters and calluses are derived from the rowing movements, and their etiology is attributed either to unconditioned hands at the start of the season or due to a change in our position. All rowing categories show the same prevalence trend irrespective of which intensity they train at.

This study also suggests that rowers tend to not let these skin injuries impact on their training or lives, and they do not stop them from rowing. Claiming that these conditions are inevitable, the rowers have adopted a coping strategy of acceptance. Apart from this coping method, the rowers also feel pride in these injuries, which also make them part of this hardy population. A practice of simple acceptance and some pride in these injuries is common. Essentially, rowers are mentally tough athletes who show that they are willing to practice their sport even at the highest level despite having blisters and calluses by adopting a cognitive mechanism to maintain control of the discomfort and pain.

Finally, it was also found that the incidence of infections in blisters may not be as high as indirectly suggested in the literature, with the consequence that one may estimate that few rowers would ever encounter any case of serious infection, either on themselves or in a fellow member of their crew, with the result that they may not fully appreciate the seriousness that such an infection, if it indeed occurs, could bring with it.

## Figures and Tables

**Table 1:** The reported number of hours spent rowing by the respondents. Responses are expressed as a percentage of population size *N* = 145). For each modality (e.g., ergometer), these responses were given to a single-choice question.

No. of Hours	Nil	0–1	1–2	2–4	4–6	6+
Ergometer/Rowing Machine	11.03	16.55	27.59	23.45	17.24	4.14
Sliding-Seat Sculling	17.93	7.59	14.48	28.28	17.24	14.48
Sliding-Seat Sweep (oar on left-hand side)	64.83	13.10	8.28	4.83	5.52	3.45
Sliding-Seat Sweep (oar on right-hand side)	61.38	15.17	10.34	7.59	3.45	2.07
Fixed-Seat/Traditional Rowing	84.83	0.69	5.52	6.21	2.07	0.69

**Table 2:** The years of experience in rowing of the respondents, expressed as actual numbers (n) and as a percentage of population size (*N =* 145). These responses were given to a single-choice question.

Do You Describe Yourself …	*n*	%
Less than 1 year	0	0
Between 1 and 3 years	11	7.59
Between 3 and 10 years	59	40.69
More than 10 years	75	51.72

**Table 3:** The self-description disclosed by the rowers at the commencement of the survey, expressed as actual numbers (n) and as a percentage of population size (*N =* 145). These responses were given to a single-choice question.

Do You Describe Yourself …	*n*	%
A rower who rows just for fun	9	6.21
A rower who trains seriously but does not compete	19	13.10
A rower who competes at Club/Regional level	61	42.07
A rower who competes at National level	47	32.41
A rower who competes at International level	9	6.21

**Table 4:** Responses, as a percentage of population size N, to the question “How long have you been rowing?” as distributed among the three categories of rowers (I, II, and III), i.e., “Fun”, “Regional/Club” and “International/National” level of competition. The responses were computed from a single-choice question.

No. of Years Rowing		<1	1–3	3–10	>10
Category I	*N =* 28	0.00	10.71	42.86	46.43
Category II	61	0.00	8.20	49.18	42.62
Category III	56	0.00	5.36	30.36	64.29
Overall Distribution	145	0.00	7.59	40.69	51.72

**Table 5:** The combined time between the different forms of rowing that the different categories of rowers spend on water, as a percentage of population size *N*. The responses were computed from a single-choice question.

Hours per Week		<1	1–2	2–4	4–6	>6
Category I	*N =* 28	0.00	3.57	50.00	21.43	25.00
Category II	61	0.00	11.30	35.48	19.35	33.87
Category III	56	0.00	9.09	30.91	20.00	40.00
Overall Distribution	145	0.00	8.97	36.55	20.00	34.48

**Table 6:** Responses, as a percentage of population size N, to the question regarding intensity of rowing during training (Intensity 1 means gently, like a warm up, 5 means very hard). The responses were computed from a single-choice question (Likert scale).

Intensity		1	2	3	4	5
Category I	*N =* 28	0.00	10.71	46.43	35.71	7.14
Category II	61	0.00	3.28	26.23	67.21	3.28
Category III	56	0.00	1.79	23.21	64.29	10.71
Overall Distribution	145	0.00	4.14	28.97	60.00	6.90

**Table 7:** Prevalence of blisters/callus as a result of rowing, reported as a percentage of population size N, the responses were computed from a single-choice question.

	Blisters				Calluses			
Category	I	II	III	Overall	I	II	III	Overall
*N*	28	61	56	145	28	61	56	145
Regularly (more than 50% of the time)	17.86	14.75	14.29	15.17	67.86	67.21	71.43	68.97
Sometimes	28.57	36.07	39.29	35.86	25.00	18.03	10.71	16.55
Occasionally/Rarely	39.29	36.07	33.93	35.86	7.14	9.84	12.50	10.34
NEVER	3.57	1.63	1.78	2.08	0.00	1.64	1.79	1.38
Only when I started rowing, not anymore	10.71	11.48	10.71	11.03	0.00	3.28	3.57	2.76

**Table 8:** Prevalence of blisters or callus as a result of non-rowing activities; the responses were computed from a single-choice question.

Category:	I	II	III	Overall
*N*	28	61	56	145
Regularly (more than 50% of the time)	0.00	0.00	0.00	0.00
Sometimes	10.71	21.31	19.64	18.62
Occasionally/Rarely	39.29	40.98	39.29	40.00
NEVER	50.00	37.71	41.07	41.38

**Table 9:** The location of blisters and calluses as reported by the rowers (expressed as %). The responses were computed from a multichoice question.

		Blisters				Calluses			
Category		I	II	III	Overall	I	II	III	Overall
	*N*	28	61	56	145	28	61	56	145
Thumb	Left	21.43	36.07	32.14	31.72	14.29	22.95	33.93	25.52
	Right	28.57	39.34	21.43	30.34	21.43	31.15	39.29	32.41
Fingers	Left	25.00	32.79	41.07	34.48	50.00	49.18	51.79	50.34
	Right	25.00	44.26	37.50	37.93	60.71	49.18	57.14	54.48
Lower Fingers/Upper Palm	Left	39.29	47.54	46.43	45.52	85.71	83.61	87.50	85.52
	Right	35.71	44.26	42.86	42.07	75.00	83.61	83.93	82.07
Palm	Left	10.71	22.95	14.29	17.24	25.00	16.39	21.43	20.00
	Right	14.29	22.95	19.64	20.00	25.00	16.39	28.57	22.76

**Table 10:** The commonly reported perceived causes of blisters as reported by the rowers. The responses were computed from a multichoice question.

Category:	I	II	III	Overall
*N*	28	61	56	145
Unconditioned hands (e.g., start of season)	50.00	55.74	50.00	52.41
When I change the oars or row in a different position	14.29	24.59	57.14	35.17
I grip too much	35.71	29.51	26.79	29.66
Over sweating	14.29	32.79	8.93	20.00
Handles are rough	7.14	9.84	3.57	6.90
I don’t know	28.57	9.84	5.36	11.72

**Table 11:** What rowers think about blisters and calluses. The responses, expressed as a percentage of the total number of respondents *N*, were computed from single-choice questions (Likert scale).

	Inevitable: Most Rowers Get Them				Painful				Ugly			
Category	I	II	III	Overall	I	II	III	Overall	I	II	III	Overall
*N*	28	61	56	145	28	61	56	145	28	61	56	145
Strongly Agree	53.57	42.62	67.86	54.48	3.57	14.75	8.93	10.34	10.71	14.75	3.57	9.66
Agree	46.43	45.90	26.78	38.62	50.00	44.26	44.65	45.52	25.00	27.87	33.93	29.65
Neutral	0.00	6.56	3.57	4.14	42.86	26.23	35.71	33.10	42.86	26.23	30.36	31.03
Disagree	0.00	1.64	1.79	1.38	3.57	9.84	7.14	7.59	21.43	19.67	19.64	20.00
Strongly Disagree	0.00	3.28	0.00	1.38	0.00	4.92	3.57	3.45	0.00	11.48	12.50	9.66

**Table 12:** The pain rating of blisters and calluses, with a pain level from not painful (1) to very painful (5). This data only considers responses of individuals who state that they actually have blisters and/or calluses. The responses, expressed as a percentage of the total number of respondents N, were computed from single-choice questions (Likert scale).

	Blisters				Calluses			
Category	I	II	III	Overall	I	II	III	Overall
*N*	24	53	49	126	28	58	53	139
1	29.17	7.55	10.20	11.03	78.57	79.31	77.36	78.42
2	25.00	24.53	24.49	21.38	10.71	15.52	13.21	13.67
3	20.83	43.40	38.78	32.41	10.71	3.45	3.77	5.04
4	16.67	16.98	22.45	16.55	0.00	1.72	5.66	2.88
5	8.33	7.55	4.08	5.52	0.00	0.00	0.00	0.00

**Table 13:** The extent of effect of blisters and calluses on different aspects of the rowers’ lives. 1 = no effect, 5 = big effect. The responses, expressed as a percentage of the total number of respondents N, were computed from single-choice questions (Likert scale).

	Effect on … Your Rowing				Effect on … Your Work/Profession				Effect on … Your Personal Life			
Category	I	II	III	Overall	I	II	III	Overall	I	II	III	Overall
*N*	28	58	53	139	28	58	53	139	28	58	53	139
1	39.29	22.41	32.08	29.50	85.71	77.05	75.00	81.29	78.57	72.13	73.21	76.98
2	28.57	50.00	47.17	44.60	7.14	11.48	16.07	12.95	14.29	18.03	14.29	16.55
3	28.57	20.69	9.43	17.99	3.57	6.56	1.79	4.32	7.14	3.28	5.36	5.04
4	3.57	3.45	11.32	6.47	3.57	0.00	1.79	1.44	0.00	1.64	1.79	1.44
5	0.00	3.45	0.00	1.44	0.00	0.00	0.00	0.00	0.00	0.00	0.00	0.00

**Table 14:** Answers to “Do you keep rowing when you have hand blisters/calluses?” (if they get them). The responses, expressed as a percentage of the total number of respondents *N*, were computed from a multichoice question.

Category	I	II	III	Overall
*N*	28	58	53	139
Yes, I have learnt to accept them	71.43	84.48	69.81	76.26
Yes, simply because I love rowing	46.43	41.38	49.06	45.32
Yes, otherwise I lose my place on the boat	0.00	3.45	3.77	2.88
Yes, but only if a competition is coming	0.00	3.45	1.89	2.16
No. I stop rowing till they heal.	0.00	1.72	1.89	1.44

**Table 15:** What rowers do to care for their blisters and calluses (if they get them). The responses, expressed as a percentage of the total number of respondents *N*, were computed from a multichoice question.

	Blisters				Calluses			
Category	I	II	III	Overall	I	II	III	Overall
*N*	24	53	49	126	28	58	53	139
Nothing	41.67	22.64	28.57	28.57	50.00	67.24	64.15	69.05
I drain them/remove them myself	29.17	45.28	51.02	44.44	14.29	18.97	16.98	19.05
I use tape to cover them	37.50	37.74	53.06	43.65	0.00	5.17	5.66	4.76
I use gloves	25.00	24.53	12.24	19.84	7.14	6.90	1.89	5.56
I use cream	8.33	9.43	12.24	10.32	14.29	15.52	15.09	16.67
Visit doctor or health/medical professional	0.00	0.00	2.04	0.79	0.00	0.00	0.00	0.00
I stop rowing for a while till they heal	4.17	7.55	4.08	5.56	0.00	0.00	0.00	0.00

**Table 16:** Rowers’ experience of blisters and calluses (if they get them). The responses, expressed as a percentage of the total number of respondents *N*, were computed from a multichoice question.

Category	I	II	III	Overall
*N*	28	58	53	139
Blisters turn into calluses	75.00	55.17	62.26	61.87
They heal on their own and I keep rowing	82.14	93.10	75.47	84.17
They only heal if/when I treat them or stop rowing	0.00	5.17	3.77	3.60
They get infected	0.00	3.45	1.89	2.16

**Table 17:** What rowers think about blisters and calluses: The concept of hard work and identity. The responses, expressed as a percentage of the total number of respondents *N*, were computed from a single-choice question.

	A Sign of Hard Work				Something to Be Proud of: They Show I Train Hard				Something to Be Proud of: They Show I AM A ROWER!!			
Category	I	II	III	Overall	I	II	III	Overall	I	II	III	Overall
*N*	28	61	56	145	28	61	56	145	28	61	56	145
Strongly Agree	7.14	13.11	17.86	13.79	3.57	14.75	16.07	13.10	10.71	18.03	17.86	16.55
Agree	28.57	31.15	16.07	24.83	17.86	16.39	17.86	17.24	39.29	24.59	33.93	31.03
Neutral	35.71	31.15	37.50	34.48	50.00	34.43	30.36	35.86	32.14	27.87	26.79	28.28
Disagree	25.00	19.67	23.21	22.07	25.00	26.23	26.79	26.21	14.29	24.59	12.50	17.93
Strongly Disagree	3.57	4.92	5.36	4.83	3.57	8.20	8.93	7.59	3.57	4.92	8.93	6.21

## Data Availability

All data is provided in the manuscript.

## References

[1] World Rowing Federation (2021). World Rowing Rule Book.

[2] Steinacker J. (2019). What Happens to the Body during a Rowing Race. https://worldrowing.com/2019/05/15/what-happens-the-body-during-rowing-race/.

[3] Anon (2016). Rowers Behaviour and the Tolerance to Pain. http://www.worldrowing.com/news/rowers-behaviour-and-the-tolerance-pain.

[4] Raja S.N., Carr D.B., Cohen M., Finnerup N.B., Flor H., Gibson S., Keefe F.J., Mogil J.S., Ringkamp M., Sluka K.A. (2020). The revised International Association for the Study of Pain definition of pain: Concepts, challenges, and compromises. Pain.

[5] O’Connor P.J., Cook D.B. (1999). 5 Exercise and Pain: The Neurobiology, Measurement, and Laboratory Study of Pain in Relation to Exercise in Humans. Exerc. Sport Sci. Rev..

[6] Anshel M.H., Williams L.R.T., Williams S.M. (2000). Coping style following acute stress in competitive sport. J. Soc. Psychol..

[7] Gandevia S.C. (2001). Spinal and supraspinal factors in human muscle fatigue. Physiol. Rev..

[8] Carver C.S., Scheier M.F., Weintraub J.K. (1989). Assessing coping strategies: A theoretically based approach. J. Pers. Soc. Psychol..

[9] Litman J.A., Lunsford G.D. (2009). Frequency of use and impact of coping strategies assessed by the COPE Inventory and their relationships to post-event health and well-being. J. Health Psychol..

[10] Ord P., Gijsbers K. (2003). Pain thresholds and tolerances of competitive rowers and their use of spontaneous self-generated pain-coping strategies. Percept. Mot. Ski..

[11] Cohen E.E.A., Ejsmond-Frey R., Knight N., Dunbar R.I.M. (2010). Rowers’ high: Behavioural synchrony is correlated with elevated pain thresholds. Biol. Lett..

[12] Davis A., Taylor J., Cohen E., Mesoudi A. (2015). Social bonds and exercise: Evidence for a reciprocal relationship. PLoS ONE.

[13] Cerasola D., Bellafiore M., Cataldo A., Zangla D., Bianco A., Proia P., Traina M., Palma A., Capranica L. (2020). Predicting the 2000-m Rowing Ergometer Performance from Anthropometric, Maximal Oxygen Uptake and 60-s Mean Power Variables in National Level Young Rowers. J. Hum. Kinet..

[14] Cataldo A., Cerasola D., Russo G., Zangla D., Traina M. (2015). Mean power during 20 sec all-out test to predict 2000 m rowing ergometer performance in national level young rowers. J. Sport. Med. Phys. Fit..

[15] Nolte V. (2011). Rowing Faster.

[16] Garland S.W. (2005). An analysis of the pacing strategy adopted by elite competitors in 2000 m rowing. Br. J. Sports Med..

[17] Muehlbauer T., Schindler C., Widmer A. (2010). Pacing pattern and performance during the 2008 olympic rowing regatta. Eur. J. Sport Sci..

[18] Thompson K.G., MacLaren D.P.M., Lees A., Atkinson G. (2004). The effects of changing pace on metabolism and stroke characteristics during high-speed breaststroke swimming. J. Sports Sci..

[19] Cerasola D., Cataldo A., Bellafiore M., Traina M., Palma A., Bianco A., Capranica L. (2018). Race profiles of rowers during the 2014 Youth Olympic Games. J. Strength Cond. Res..

[20] Steinacker J.M. (1993). Physiological aspects of training in rowing. Int. J. Sports Med..

[21] Karlson K.A. (2000). Rowing injuries. Phys. Sportsmed..

[22] Rumball J.S., Lebrun C.M., Di Ciacca S.R., Orlando K. (2005). Rowing injuries. Sport. Med..

[23] Knapik J.J., Reynolds K.L., Duplantis3 K.L., Jones B.H. (1995). Friction Blisters Pathophysiology, Prevention and Treatment. Sports Med..

[24] Brennan F.H. (2002). Managing Blisters in Competitive Athletes. Curr. Sports Med. Rep..

[25] Tlougan B.E., Podjasek J.O., Adams B.B., Perelman R.O., Brook Tlougan C.E. (2010). Aquatic sports dermatoses: Part 3 On the water. Int. J. Dermatol..

[26] Monique Mokha G., Hauck S. (2014). Dermatoses in rowers. Int. J. Athl. Ther. Train..

[27] Levine N. (1982). Friction blisters. Phys. Sportsmed..

[28] Adams B.B. (2002). Dermatologic Disorders of the Athlete. Sports Med..

[29] Mailler E.A., Adams B.B. (2004). The wear and tear of 26.2: Dermatological injuries reported on marathon day. Br. J. Sports Med..

[30] Nicholl J.P., Coleman P., Williams B.T. (1995). The epidemiology of sports and exercise related in jury in the United Kingdom. Br. J. Sport. Med..

[31] Smith J., Krabak B. (2002). Tug of war: Introduction to the sport and an epidemiological injury study among elite pullers. J. Med. Sci. Sport..

[32] Harmer P.A., Rocha Piedeade S., Neyret P., Espregueira-Mendes J., Cohen M., Hutchinson M.R. (2021). Fencing epidemiology and organizational prophylaxis. Specific Sports-Related Injuries.

[33] Majorano M., Cesario S. (1991). Lesioni traumatiche acute nella pratica sportive agnostica della scherma. Med. Sport..

[34] Roi G.S., Fasci A. (1988). Indagine sulle richieste di intervento del medico durante le gare di scherma. Ital. J. Sport Traumatol..

[35] Feher R. (2009). The Epidemiology of Injuries Sustained by Canoeists during the 2006 Isuzu Berg River Canoe Marathon. Master’s Thesis.

[36] Mukherjee S., Leong H.F., Chen S., Foo Y.X.W., Pek H.K. (2014). Injuries in competitive dragon boating. Orthop. J. Sport. Med..

[37] Wyatt R. (2013). Rowing Blisters and Skin Injuries.

[38] Bruce A. (2014). Care and Feeding of Rowing Blisters—Swiss Cheese Hands. https://www.row2k.com/features/789/Swiss-Cheese-Hands--the-care-and-feeding-of-rowing-blisters/.

[39] (2017). World Rowing the Blister Dilemma and How to Care for Them. https://worldrowing.com/2017/01/10/the-blister-dilemma-and-how-care-for-them/.

[40] Lee L. How to Heal Blisters from Rowing. www.wikihow.com/Heal-Blisters-from-Rowing.

[41] Creswell W.J., Creswell J.D. (2018). Research Design: Qualitative, Quantitative adn Mixed Methods Approaches.

[42] Scotland J. (2012). Exploring the philosophical underpinnings of research: Relating ontology and epistemology to the methodology and methods of the scientific, interpretive, and critical research paradigms. Engl. Lang. Teach..

[43] Pring R. (2000). The ‘False Dualism’ of Educational Research. J. Philos. Educ..

[44] Nick T.G., Ambrosius W.T. (1987). Descriptive Statistics. Methods in Molecular Biology.

[45] Hoffman M.D., Krouse R. (2018). Ultra-obligatory running among ultramarathon runners. Res. Sport. Med..

[46] Lazarus R.S., Folkman S. (1984). Stress, Appraisal, and Coping.

[47] Roebuck G.S., Urquhart D.M., Knox L., Fitzgerald P.B., Cicuttini F.M., Lee S., Fitzgibbon B.M. (2018). Psychological factors associated with ultramarathon runners’ supranormal pain tolerance: A pilot study. J. Pain.

[48] Simpson D., Post P.G., Young G., Jensen P.R. (2014). “It’s not about taking the easy road”: The experiences of ultramarathon runners. Sport Psychol..

[49] Tesarz J., Schuster A.K., Hartmann M., Gerhardt A., Eich W. (2012). Pain perception in athletes compared to normally active controls: A systematic review with meta-analysis. Pain.

[50] Costa J., Pinto-Gouveia J. (2011). Acceptance of pain, self-compassion and psychopathology: Using the Chronic Pain Acceptance Questionnaire to identify patients’ subgroups. Clin. Psychol. Psychother..

[51] Morgan W.P., Pollock M.L. (1977). Psychologic characterization of the elite distance runner. Ann. N. Y. Acad. Sci..

[52] Stevinson C.D., Biddle S.J. (1998). Cognitive orientations in marathon running and “hitting the wall”. Br. J. Sports Med..

[53] Coons M.J., Steglitz J. (2013). Pain threshold. Encyclopedia of Behavioral Medicine.

[54] Scott V., Gijsbers K. (1981). Clinical Pain perception in competitive swimmers. Br. Med. J..

[55] IASP (1994). IASP Terminology—IASP. In *Classification of Chronic Pain*, 2nd ed.; IASP Task Force on Taxonomy. https://www.iasp-pain.org/resources/terminology/.

[56] Crust L. (2008). Mental Toughness and Coping in an Ultra-Endurance Event.

[57] Jones G. (2002). What is this thing called mental toughness? An investigation of elite sport performers. J. Appl. Sport Psychol..

[58] Jones G., Hanton S., Connaughton D. (2007). A framework of mental toughness in the world’s best performers. Sport Psychol..

[59] Thelwell R.C., Greenlees I.A. (2003). Developing Competitive Endurance Performance Using Mental Skills Training. Sport Psychol..

